# Thinning increases forest resiliency during unprecedented drought

**DOI:** 10.1038/s41598-022-12982-z

**Published:** 2022-05-31

**Authors:** Temuulen Sankey, Julia Tatum

**Affiliations:** School of Informatics, Computing, and Cyber Systems, Northern Arizona University, 1295 S. Knoles Drive, Flagstaff, AZ 84011 USA

**Keywords:** Climate change, Environmental impact

## Abstract

Regional droughts are now widespread and are projected to further increase. Semi-arid ponderosa pine forests across the western USA, which occupy > 56 million ha, are experiencing unprecedented levels of drought due to the currently ongoing North American megadrought. Using unpiloted aerial vehicle (UAV) thermal images and ground-based hyperspectral data, here we show that ponderosa pine forest canopy temperatures increased during the 2021 summer drought up to 34.6 °C, far above a typical canopy temperature when ponderosa pine trees no longer uptake carbon. We infer that much of the western US ponderosa pine forests likely served as a net carbon source rather than a sink during the 2021 summer drought period. We also demonstrate that regional forest restoration thinning significantly reduced the drought impacts. Thinned ponderosa pine forests had significantly lower increase in canopy temperature and canopy water stress during the drought period compared to the non-thinned forest stands. Furthermore, our extensive soil moisture network data indicate that available soil moisture in the thinned forest was significantly greater at all soil depths of 25 cm, 50 cm, and 100 cm compared to the non-thinned forest, where soil moisture dry-down in the spring started significantly earlier and stayed dry for one month longer causing critical water stress for trees. Forest restoration thinning benefits that are otherwise unappreciated during average precipitation years are significantly amplified during unprecedented drought periods.

## Introduction

Regional droughts are becoming hotter, drier, and more frequent due to global climate change and temperature warming^1–3^. Semi-arid ecosystems, which cover ~ 18% of the earth’s surface^4^, are projected to be among the most vulnerable to increased drought^5^. Semi-arid ecosystems are also the primary drivers of interannual variability in atmospheric CO_2_ fluxes^6,7^. Changes in semi-arid forest structure and function can, therefore, have global implications. It is important to understand the interactions between regional drought and semi-arid forest function because drought is further projected to get increasingly common^5^.

**Figure 1:**
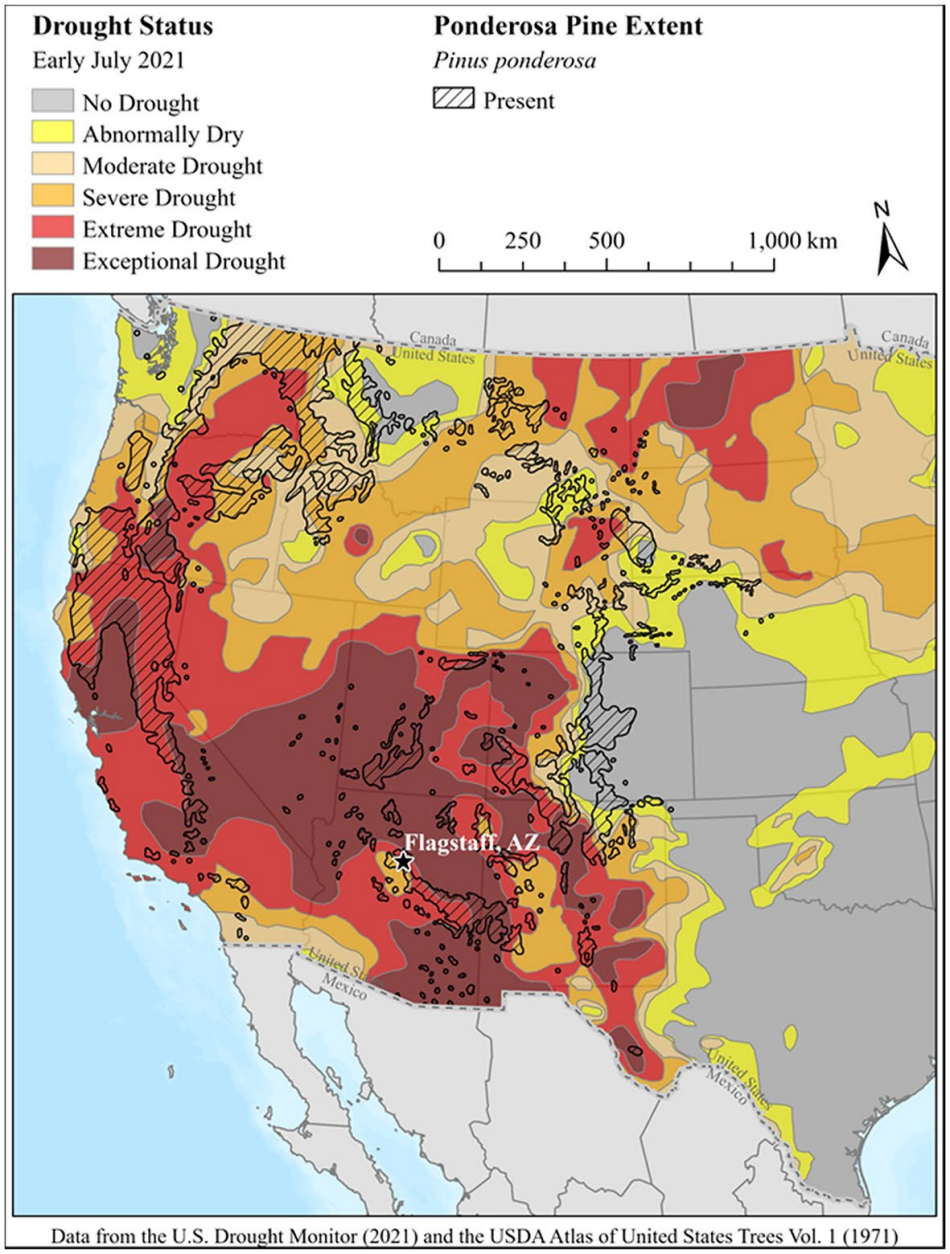
Drought status across the western US in July, 2021 (GIS data from Little, 1971, and US Drought Monitor, 2021).

Drought-induced forest water stress, sometimes coupled with insect impacts, can cause large-scale tree mortality and growth declines^1,8–10^. In ponderosa pine (*Pinus ponderosa)* forests, which span over 56 million hectares across the western US (Fig. 1), this results in significant reductions in tree density, mean growth, tree diameter, and basal area^11,12^. In summer 2021, 44% of ponderosa pine forests across the western US experienced “extreme” and “exceptional” drought levels, while another 39% experienced “moderate” to “severe” drought conditions (Fig. 1).

**Figure 2:**
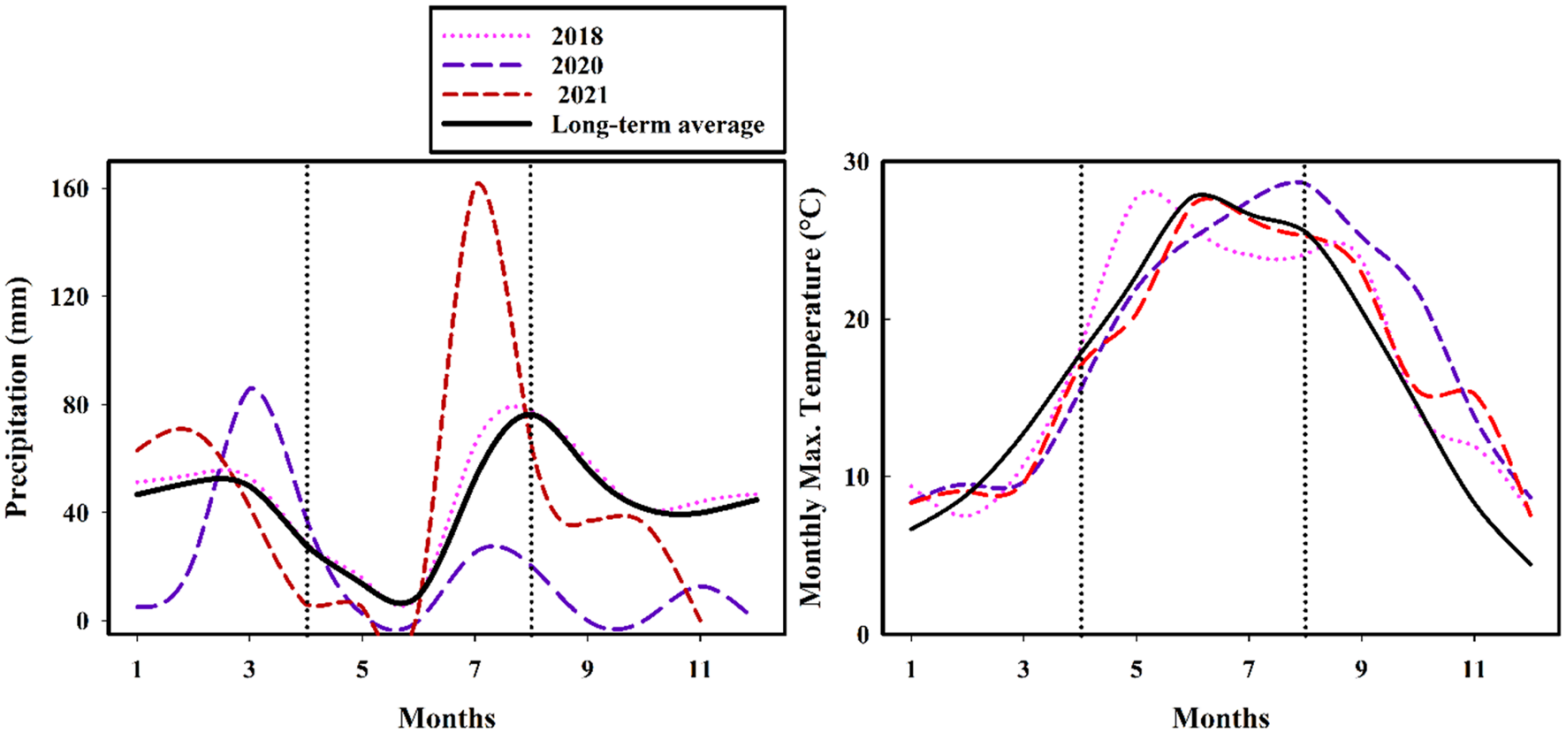
Precipitation and monthly maximum temperature in average year 2018 and drought years 2020 and 2021 compared to the long-term mean precipitation in northern Arizona. During the megadrought, late July 2021 experienced two record rainfall events (100-year event followed by a 200–500-year event), which resulted in flash floods in northern Arizona, although precipitation remained well below the long-term mean precipitation during our study prior to the flood events since April 2020.

The currently ongoing North American megadrought^13^ has had the most notable impacts in the semi-arid southwestern US (Fig. 1). We quantify its effects on ponderosa pine forest canopy temperature, canopy moisture, and soil moisture using in-situ and remote sensing data in northern Arizona, which experienced two consecutive dry years in 2020 and 2021 that created the “extreme” and “exceptional” drought conditions, in particular since late April, 2020 (Fig. 2) (NOAA weather station).

Using high spatial resolution unpiloted aerial vehicle (UAV) thermal images from an average precipitation year 2018 and during the record dry conditions in 2021, we quantify drought-induced canopy temperature changes in ponderosa pine forests in a northern Arizona study site (83 ha) at a local scale. UAV-based rapid and frequent assessments at local scales might also provide potential early indicators of large-scale forest mortality^9,12^ often associated with drought^14,15^.

Remote sensing of forest canopy temperatures is critical in understanding drought impacts on ecosystem function because canopy temperature in arid and semi-arid environments reflects critical plant physiological processes, particularly canopy water content, transpiration, and C fluxes^16–18^. Many tree species maintain canopy temperature close to the ambient temperature via transpiration and evaporative cooling^19,20^, which requires readily available soil water. In hotter, drier drought periods, less-available soil water reduces canopy transpiration and evaporative cooling, which can lead to hotter canopy temperatures and reduced C uptake^21^. Forest remote sensing at a landscape scale provides rapid, accurate, and accessible measurements of canopy temperature and canopy moisture content changes in response to regional drought^18,22^.

Forest restoration thinning can improve drought resiliency by increasing summer-time canopy moisture content for the remaining trees and increasing winter snow accumulation on the ground^22–25^. Forest managers are now considering restoration treatments at larger scales to reduce vegetation water stress and improve soil moisture and drought resiliency^26,27^. The Four Forest Restoration Initiative (4FRI) implemented across the state of Arizona by the US Forest Service is the first and largest example of such restoration efforts, although its primary goal is to reduce catastrophic wildlife risks. 4FRI intends to treat over one million hectares of forests statewide via thinning and burning. We combine our UAV thermal images with ground-based hyperspectral spectroradiometer data to quantify 4FRI forest thinning effects on drought-impacted forest canopy temperature and canopy moisture content. Both canopy water content and temperature impact forest biogeochemical processes such as C fluxes and are important to quantify especially during drought^28^. Furthermore, hyperspectral remote sensing measurements of canopy moisture content provide a direct explanation for potential changes observed in canopy temperature during drought because canopy moisture content and transpiration rates largely drive canopy temperature^16,17^.

Forest restoration thinning can also impact available soil moisture. Regional droughts reduce soil moisture in the vegetation rooting zone^14,29^ and can lead to landscape-scale tree mortality^30^. Soil moisture in thinned forests has been documented to be greater than in non-thinned forests in some studies during average precipitation years^31–33^, while other studies demonstrate further benefits during a drought year^34^. 4FRI restoration provides an opportunity to examine the thinning impacts on soil moisture during “extreme” or “exceptional” drought periods.

Using an unprecedented soil moisture dataset from 93 sensors at the northern Arizona study site (83 ha), we compare soil moisture during the regional “extreme” drought period in thinned versus non-thinned dense forests at soil depths of up to 100 cm at hourly temporal resolution. Previous studies have documented forest thinning effects on soil moisture in average precipitation years using much fewer soil moisture sensors at shallow depths of up to 40 cm in the soil profile^33^. Our extensive soil moisture network allows a detailed evaluation of below-ground moisture content changes due to drought. In contrast, the more readily available remote sensing measurements provide an estimate of above-ground canopy temperature and canopy moisture content changes in response to the drought and potential changes in soil moisture. Taken together, the below-ground measurements of soil moisture and above-ground hyperspectral measurements of forest canopy moisture, and UAV-based canopy temperature measurements provide a comprehensive assessment of ecosystem response to drought.

## Results and discussion

### Drought impacts on forest canopy temperature and moisture

We measured post-thinning forest canopy temperature (°C) on August 10, 2018, an average precipitation year, and on July 9, 2021 during the peak of the regional drought period (Fig. 2). While these measurements are approximately a month apart in the two years, July and August in normal years have relatively similar mean temperatures at 20 °C and 19 °C, respectively. Our results highlight three important findings: (1) restoration thinning increases forest canopy temperature and evaporative cooling demand across years, (2) canopy temperatures during the drought year 2021 increased to extremely high temperatures of 34.6 °C, when trees likely served as a net carbon source rather than a sink, and (3) canopy temperature increase during drought year 2021 was significantly greater in non-thinned, dense forest than in the thinned forest making the dense forest much more vulnerable to drought.

**Figure 3:**
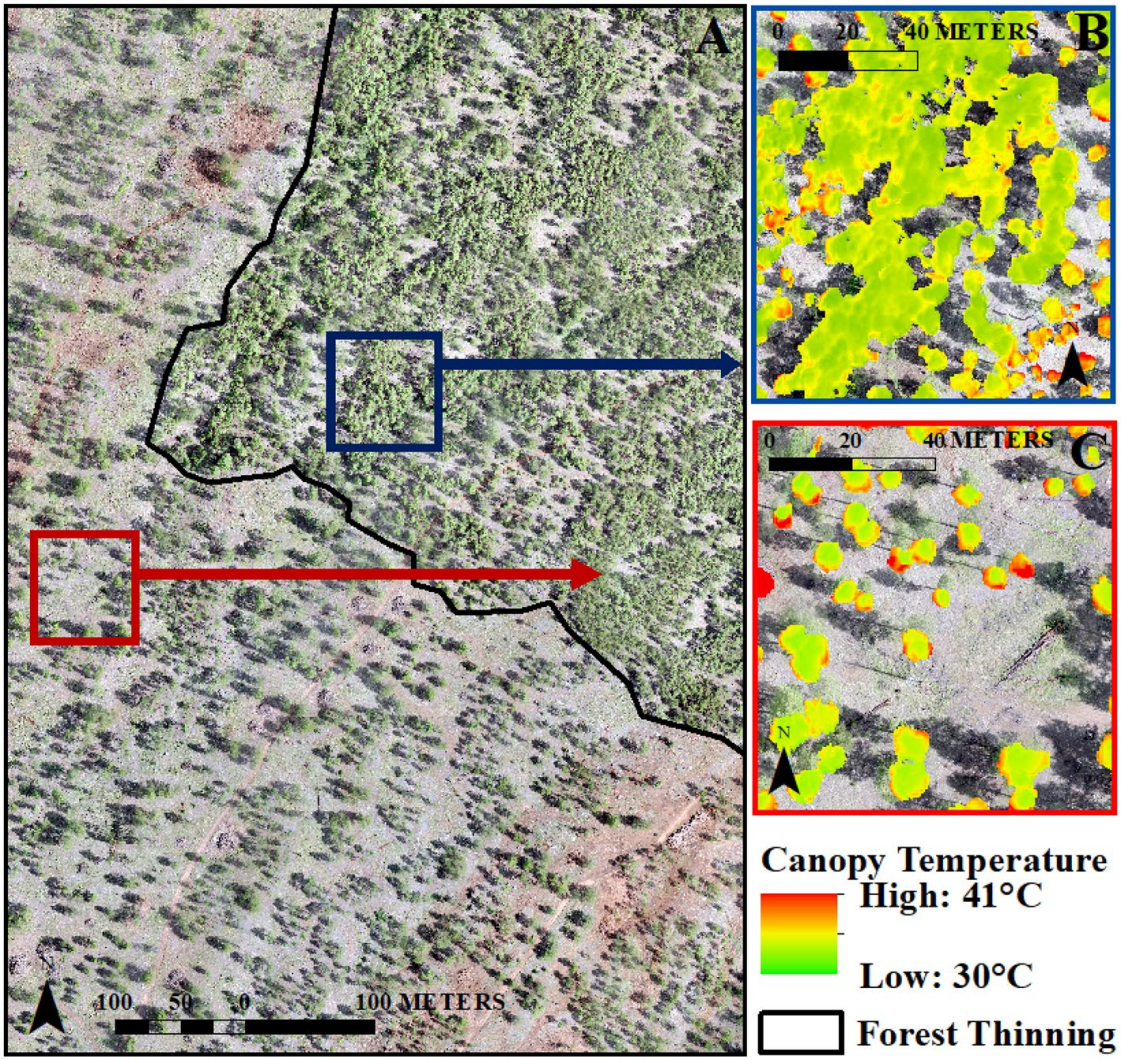
UAV image across a forest thinning boundary at the study area (panel **A**) and UAV thermal image-derived ponderosa pine forest canopy temperature examples from non-thinned (panel **B**) and thinned (panel **C**) forests in northern Arizona during drought year 2021.

First, average canopy temperature varied substantially by forest density conditions in 2018 under normal precipitation conditions (mean = 25.8 °C degrees (SD = 3.16 °C)) (Fig. 3). The mean canopy temperature in the thinned forest stand was significantly greater at 28.1 °C (SD = 3.6 °C) (p < 0.0001) compared to the 24.8 °C mean temperature (SD = 2.1 °C) in the non-thinned forest stand during the average precipitation year. This is consistent with previous studies that documented strong relationships between canopy cover and temperature^18,20,35^. Specifically, the restoration thinning had created many small patches of trees and individual trees that were isolated and sparse, which showed high canopy temperature values in 2018. In contrast, larger forest patches in the thinned forest as well as the dense, non-thinned forest had cooler canopy temperature values similar to previously reported ponderosa pine canopy temperature ranges^20,21^. This suggests that forest canopy temperatures are hotter following restoration thinning and thus trees in thinned areas have greater evaporative cooling demand than the trees in non-thinned areas.

Secondly, the mean canopy temperature during the drought year 2021 across the entire study area was extremely high at 34.6 °C degrees (SD = 0.86 °C) (Fig. 2). Such increases in canopy temperature indicate drought stress^16,36^ and reduced canopy transpiration and sap flow^35^. During drought periods, plants close their stomata and reduce their transpiration rates, which, in turn, results in increased canopy temperature and reduced carbon uptake. The observed high canopy temperature in Arizona likely occurred throughout much of the ponderosa pine forest across the western US because > 25 million hectares of western US ponderosa pine forests had “extreme” and “exceptional” levels of drought in 2021 (Fig. 1).

Importantly, canopy temperature is tightly linked to C exchange in ponderosa pine forests and net ecosystem exchange has a stronger correlation with canopy temperature than air temperature, as documented in semi-arid central Oregon, USA with warm, dry summers and cool, wet winters^21^, which has similar total annual precipitation to northern Arizona and where ponderosa pine forests also experienced “extreme” and “exceptional” drought in 2021 (Fig. 1). Ponderosa pine trees uptake C when canopy temperature is between 5 and 20 °C, but they release C to the atmosphere when canopy temperatures exceed 30 °C because trees at such high temperatures become water-stressed^21^. In summer 2021, canopy temperatures across our study area ranged between 31 and 41 °C and even the minimum value was greater than 30 °C. If this range was observed across the entire 25 million hectares of “extreme-exceptional” drought area (Fig. 1), these ponderosa pine forests likely served as a net C source during the daytime for much of summer 2021, since canopy temperatures impact photosynthesis and leaf respiration.

**Figure 4:**
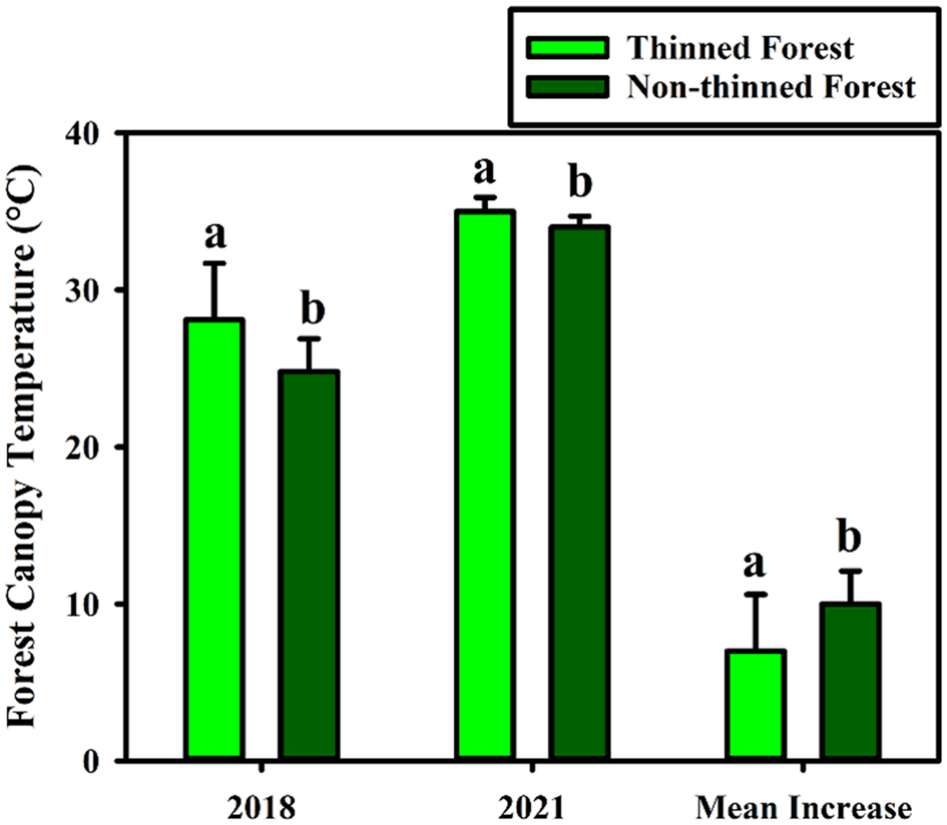
UAV thermal image-derived mean canopy temperature in thinned (N = 10,000 randomly-selected pixels) and non-thinned (N = 10,000 randomly-selected pixels) ponderosa pine forests during average precipitation year 2018 and drought year 2021. The different letters within pairs of bars (a versus b) indicate a statistically significant difference (p < 0.01). Thinned forest canopy temperature is consistently and significantly (p < 0.001) greater when compared to non-thinned forest canopy temperature using an ANOVA test. However, mean canopy temperature increase from average precipitation year 2018 to drought year 2021 is significantly (p < 0.001) greater for the non-thinned forest compared to the thinned forest.

In summer 2021, the thinned forest still had a significantly greater mean canopy temperature compared to the non-thinned forest (*p* < 0.0001) (Fig. 4). Interestingly, as the forest canopy temperature across the entire study site increased during the drought year, forest canopy temperatures between the thinned versus non-thinned forest stands became much more similar. Trees in the non-thinned, dense forest were well buffered during the average precipitation year, but also experienced extremely high temperatures during the drought year similarly to the isolated individual trees.

Third, our results indicate that the non-thinned, dense forest stands are significantly more vulnerable to severe drought conditions, although these stands are cooler overall. The non-thinned forest stand had a significantly greater increase of 9.5 °C (SD = 2.1 °C) in canopy temperature compared to a 6.9 °C increase (SD = 3.6 °C) at the thinned forest stand (Fig. 4). The greater canopy temperature increase in the non-thinned forest is likely due to competition among the dense trees for available soil water, which outweighs the shading effects that they provide for each other during normal years. Restoration thinning reduces such competition and better buffers the remaining trees during drought years, although mean canopy temperatures in the thinned areas might be consistently high across years. Forest restoration thinning can, therefore, be used as a tool to reduce water demand at the stand level and increase drought resiliency^37^.

**Figure 5:**
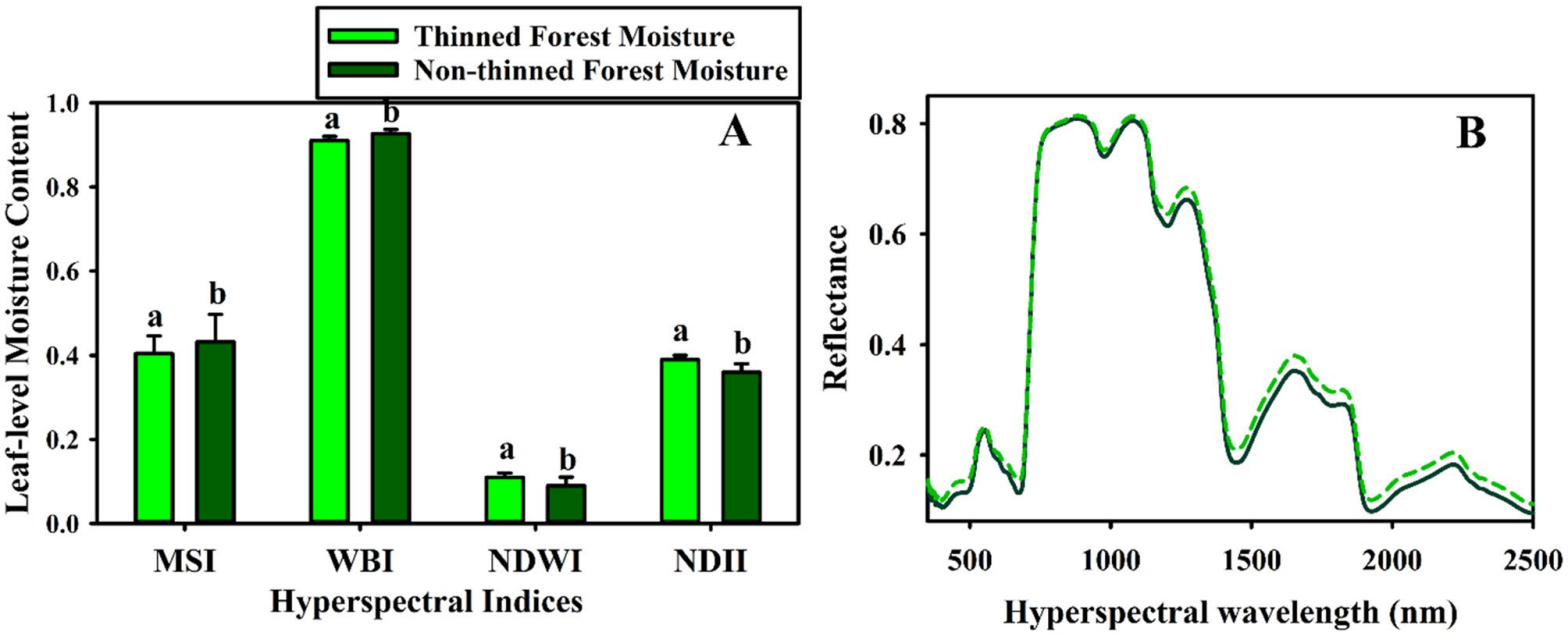
Hyperspectral indices of forest moisture content (Panel **A**) and hyperspectral reflectance (Panel **B**) from tree samples in thinned (N = 31) and non-thinned (N = 33) forests during 2021 drought year. The bars and whiskers indicate the mean values and standard errors for each index, respectively. The different letters (a versus b) within pairs of bars indicate statistically significant differences (p < 0.05). MSI and WBI are simple ratios, which increase with greater plant water stress. NDWI and NDII are normalized difference ratios that decrease as plant water content decreases. When compared with ANOVA tests, all four hyperspectral indices indicated that the non-thinned, dense forest trees have significantly lower plant water content (all p < 0.05) and are significantly more water-stressed compared to the thinned forest trees.

Our results on the differences in canopy-level temperature increase were also consistent with leaf-level hyperspectral data, which indicated significantly lower moisture content among the densely distributed trees in the non-thinned forest compared to the thinned forest (all p < 0.05) in four different moisture indices (Fig. 5). This result provides a direct explanation for the significantly greater increase in the non-thinned forest canopy temperature because canopy temperature increases as canopy moisture content decreases^21^. This also suggests that the competition for available soil water among the dense trees in the non-thinned forest make these trees particularly water-stressed and vulnerable to drought impacts^37^.

Our results suggest that restoration thinning impacts on forest canopy temperature are significant and particularly beneficial during severe drought years for ecosystem resiliency and net ecosystem exchange. Importantly, these benefits do not appear significant or might show the opposite trends in canopy temperature during average years. However, the benefits are significant during drought years and will be especially important in addressing climate change impacts in the coming years. Hotter and drier droughts are projected to increase and will likely trigger further impacts from insects and diseases in forested ecosystems^12,38–40^. While the main goal of the regional forest restoration efforts in Arizona and other western states is to reduce catastrophic wildfire impacts^22,26,27^, our results highlight the benefits of the restoration thinning treatment during exceptional and extreme drought periods. Our data illustrate the patterns of increased canopy temperature, greater evaporative cooling demand, and lower moisture content that occur in dense forest stands^37,38,41,42^, which ultimately lead to drought-induced forest mortality.

### Drought impacts on forest soil moisture

Another key driver of drought-induced forest mortality around the world is soil water availability [15; 30], which impacts forest canopy water content^43^. Our continuous, hourly measurements from 93 soil moisture sensors reveal three important findings: (1) soil moisture is significantly lower in the non-thinned, dense forest at all depths during the drought period compared to the thinned forest, (2) spring soil moisture dry-down starts significantly earlier (29 days earlier) in the non-thinned forest, which then experiences much longer dry periods, compared to the thinned forest, and (3) soil moisture availability shows consistent trends with forest canopy moisture content and temperature estimates.

**Figure 6:**
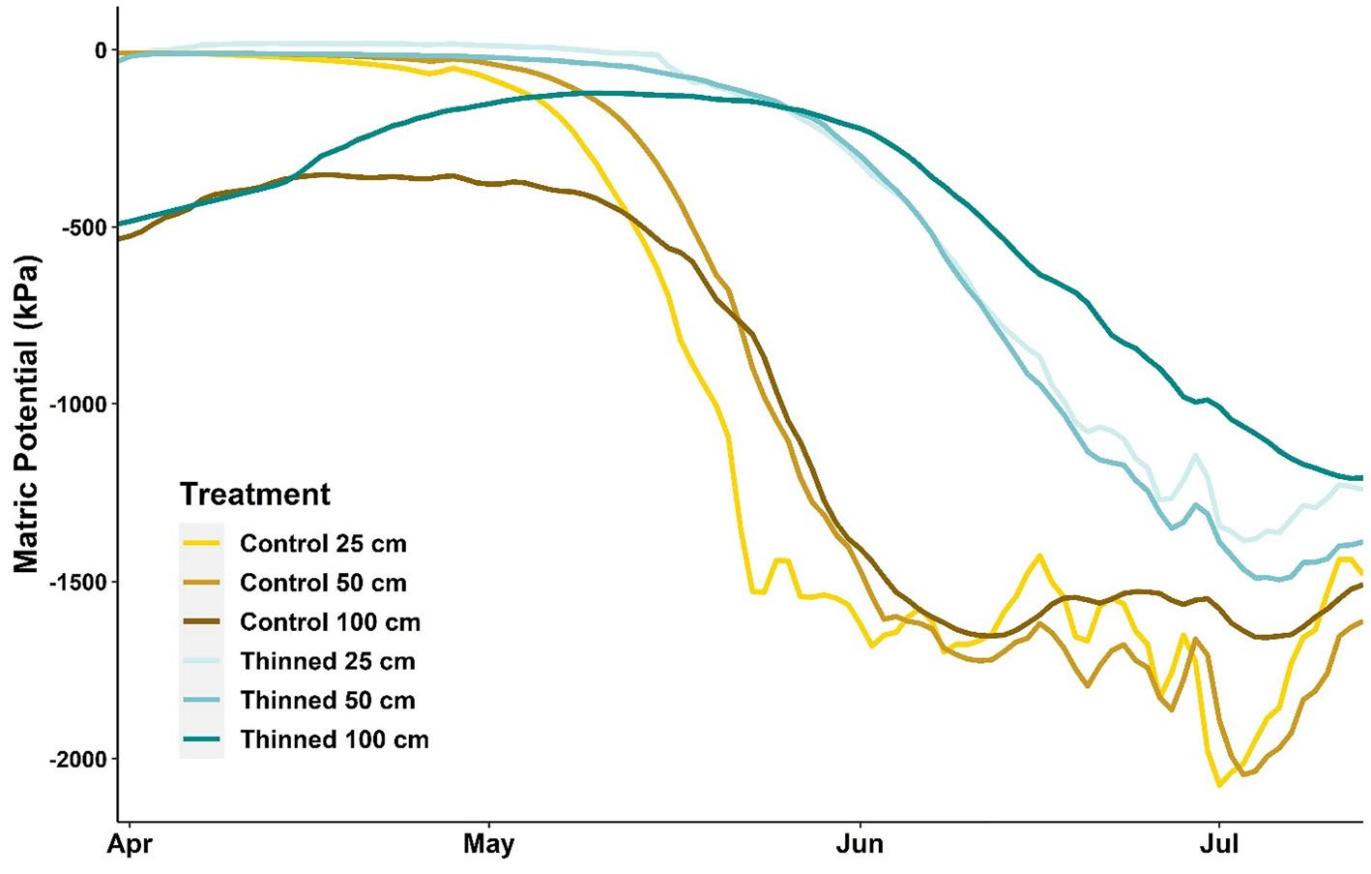
Soil moisture trends at 25 cm, 50 cm, and 100 cm depths in the thinned and non-thinned control forest during the summer 2021 drought period.

First, average soil water potential across all depths was significantly lower in the dense, non-thinned forest at −913 kPa (SD =  ± 233) compared to −498 kPa (SD = 318) in the thinned forest during the 2021 drought period (*p* < 0.001) (Fig. 6). Consistent with the canopy temperature results above, this indicates that the non-thinned, dense stands face greater vulnerability to regional drought likely due to competition among the dense trees for available soil water. In contrast, the thinned forest sustained greater soil moisture availability for the remaining trees. Our extensive soil moisture network previously indicated significantly drier soils in areas of higher basal area, tree density, and canopy cover at shallower depths during relatively dry year of 2019 and drought year 2020^34^. This trend was further amplified during the 2021 exceptional drought across all soil depths, when significant differences in soil moisture became consistently observed between thinned versus non-thinned forests across a forest density gradient (Fig. 6). Taken together, these results highlight the benefits of forest thinning that are not significant during average precipitation years but become critical during exceptionally dry periods.

Importantly, soil water potential at the 100 cm depth was significantly lower in the non-thinned, dense forest compared to the thinned forest. While only a few of our sensors are located in the non-thinned forest compared to the thinned forest, this result is particularly alarming given that soil moisture at 100 cm is the major water source for trees and buffers them from drought-induced water stress and eventual tree mortality^33^. Furthermore, significant differences in soil moisture during average precipitation years are typically observed only at shallower depths^32,44,45^, and rarely at depths up to 100 cm^34,46^. The extremely dry conditions in summer 2021 had a far-reaching impact down to 100 cm in the soil profile. Interestingly, the forest thinning treatment significantly impacted this trend providing a strong buffer for the thinned forests. Although it is a challenge to install and maintain a large number of soil moisture sensors equally distributed between thinned and non-thinned forests, especially at 100 cm depth, future studies need to monitor this trend over larger areas.

Secondly, soil moisture dry-down at all depths in the spring 2021 after snowmelt started significantly earlier (29 days earlier) in the non-thinned forest compared to the thinned forest, which further supports our previous finding that the higher levels of basal area, tree density, and canopy cover all translated to a significantly earlier onset of soil drying^34^. We further observed soil moisture dry-down trends until the soil water matric potential fell below a −1000 kPa critical drying threshold, which induces substantial water stress for ponderosa pine trees^34^. During the 2021 drought, the non-thinned forest experienced 29 more days below this critical drying threshold compared to the thinned forest (*p* < 0.001). Importantly, our previous research documented that the ponderosa pine forest study site in northern Arizona did not reach the critical drying threshold in 2019, especially at 100 cm, but the drought year 2020 had 7.7 days below this threshold across the entire study site^34^, whereas the 2021 drought year resulted in 29 days below the critical drying threshold as the drought condition prolonged across the region. These results further support previous trends of deep soils drying early and staying dry for longer periods due to climate change^47^. It is likely that much of the 56 million ha of ponderosa pine forest across the US experienced soil moisture levels below this critical threshold during the 2021 drought period because 84% of these forests experienced “moderate” to “exceptional” levels of drought (Fig. 1). It is also likely that these forests experienced similar levels of soil water stress during summer 2020, which also experienced lower than average precipitation due to the current megadrought.

Finally, our soil moisture results consistently underscore the trends observed in our canopy temperature and moisture content estimates: (1) the current megadrought is associated with extensive moisture stress in ponderosa pine forest canopies as well as forest soils throughout the soil profile, and (2) the moisture stress observed in the thinned forest was significantly less severe than in the non-thinned, dense forests. These results indicate that the non-thinned, dense forests are significantly more vulnerable to drought than thinned forests. Furthermore, similar water stress both below- and above-ground was likely imposed on ponderosa pine forests across the western US as they experienced exceptional levels of drought in summer 2021. Such water stress, if sustained, will likely result in regional-scale forest mortality. Importantly, large-scale forest restoration efforts are significantly reducing extreme drought impacts and improving ecosystem resiliency to drought.

## Methods

### Study setting

**Figure 7:**
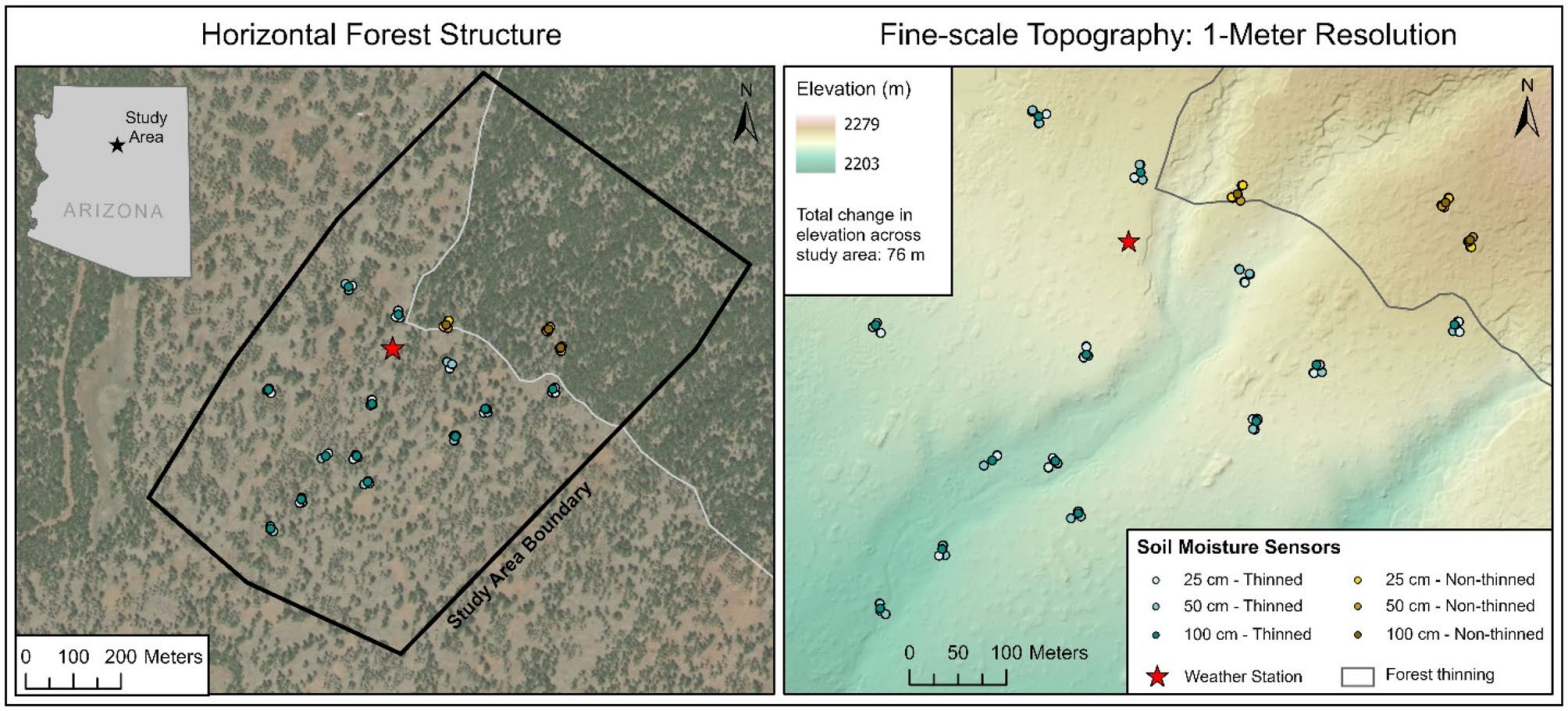
Study area (83 ha, outlined with a black line in the left panel) in northern Arizona that was thinned as part of the regional forest restoration effort. The soil moisture sensor network includes 93 sensors that measure hourly soil water potential and a zoomed-in view of their distribution is shown in the right panel. The entire 83 ha study area (outlined with a black line in the left panel) was imaged with the UAV thermal sensor in average precipitation year 2018, but a smaller extent was imaged during the 2021 exceptional drought period due to the National Forest closure because of fire risk concerns.

The ponderosa pine forest study area is located in the Coconino National Forest in northern Arizona, USA. At 2200–2275 m elevation, the study area has relatively flat topography (0–10% slopes) (Fig. 7). Northern Arizona has a sub-humid climate with 560 mm of average annual precipitation (Fig. 2). The climate is characterized by strong seasonal trends including a winter snow, early summer drought, and late-summer monsoonal seasons (http://www.wrcc.dri.edu). The summer monsoon is usually in July and August, when mean monthly precipitation is 52 mm and 72 mm, respectively (Fig. 2). Mean monthly temperatures for July and August are typically very similar at 20 °C and 19 °C.

The ponderosa pine *(Pinus ponderosa)* vegetation includes occasional Gambel oak *(Quercus gambelii)* and Rocky Mountain juniper *(Juniperus scopulorum)*. Arizona fescue (*Festuca arizonica*), mountain muhly (*Muhlenbergia montana*), mutton bluegrass (*Poa fendleriana*), bottlebrush squirreltail (*Sitanion hysterix*), and Buckbush (*Ceanothus fendleri*) comprise the understory vegetation, which is typical of the region’s ponderosa forest. The study area was burned in a naturally occurring wildfire in 1876, and a prescribed fire in 1976 eliminated 63% of the smaller surface fuels and 69% of the larger woody surface fuels (up to 8 cm in diameter)^48^.

A mechanical thinning treatment was implemented across the study area during 2017–2018 as a part of the regional 4FRI effort^48^. Our monitoring started at this study site prior to the thinning treatment^48^. The study site includes a 53 ha thinned forest adjoining a 30 ha non-thinned forest, which initially included 23 ha but was recently expanded (Figs. 2 and 7). The regional thinning treatment is aimed at reducing the risk of catastrophic wildfire by re-creating the less dense pre-settlement forest conditions and promote healthy overstory vegetation and the regeneration of understory vegetation. The thinning treatment at this study area prescribed creating irregular tree groups, increasing overall interspace, retaining all non-ponderosa pine species, and significantly reducing the number of smaller ponderosa pine trees. These objectives were successfully met: (a) forest canopy cover was reduced from 39.4 to 9.6%, (b) stem density was reduced from 212.4 to 64.5 trees per ha, (c) the corresponding basal area was reduced from 22.9 to 13.2 m^2^/ha, (d) the treatment created considerable canopy openings by breaking up once continuous forest patches, often leaving single trees or groups of 2–3 trees behind, (e) treatment increased the number of patches by 70.6%, and decreased the mean patch area by 80.8% to 0.13 ha, and (f) the total patch area across the study site decreased by 39.6% with a corresponding 74% increase of interspace area^48^.

### Forest canopy temperature estimates

Forest canopy temperature (°C) was estimated in 25 cm spatial resolution using a Sensefly eBee RTK fixed-wing UAV platform equipped with a thermoMAP thermal sensor on August 10, 2018, an average precipitation year, and on July 9, 2021 during the peak of the regional drought period. The UAV flights were completed close to solar noon on each day with similar air temperatures (26–30 °C) and wind speed (< 2 km/h) at 90 m altitude aboveground with 85% and 90% latitudinal and longitudinal overlaps, respectively. Flight mission planning and data parameters were customized and executed in SenseFly eMotion 3 software (SenseFly, Lausanne, Switzerland). The thermoMAP sensor was internally calibrated during each flight between images and has a temperature resolution of 0.1 ºC across a range of -40 ºC degrees to 160 ºC degrees (SenseFly, Lausanne, Switzerland). The resulting images, therefore, are seamlessly mosaicked without additional calibration pre- or post-flight (SenseFly, Lausanne, Switzerland). Similar to previous studies^49–53^, the geolocation errors reported by the SenseFly eMotion software and our field-based GPS validation indicated errors of up to 1.7 m in the X, Y, and Z dimensions^48^.

The two temperature images were first co-registered (RMSE = 20 cm) and overlaid to a canopy height model image^48^, which was generated using a Structure-from-Motion algorithm within the Pix4D software (SenseFly, Lausanne, Switzerland) and a multispectral image acquired using the Sensefly eBee fixed-wing UAV with a Sequoia multispectral sensor (SenseFly, Lausanne, Switzerland). Only the forest canopy temperature pixels that overlapped with tree canopy heights ranging 2-40 m were extracted. There is no shrub understory. It was important to exclude the herbaceous understory vegetation and bare soil temperature pixels, which had much greater temperatures than tree canopies, which were the focus of this study. Individual tree canopies were identified and delineated. Then, the mean temperature for each tree canopy (N =  ~ 650 trees) was calculated for each image date within thinned and non-thinned forest stands. The mean canopy temperature values as well as the differences were then compared between the drought year 2021 and the average precipitation year 2018 in thinned and non-thinned forest stands using ANOVA tests and a total of 20,000 random pixel samples from within the tree canopies. Of these, 10,000 random pixel samples were distributed within the tree canopies in the thinned forest, whereas the other 10,000 random pixels were in the non-thinned forest. Prior to the ANOVA tests, we generated boxplots and tested for normal distributions in the data using Levene’s test.

### Forest canopy moisture estimates

Hyperspectral spectra were measured within both thinned and non-thinned ponderosa pine forests on May 28, 2021 and June 1, 2021 during the drought period using a hand-held ASD Inc. FieldSpec 3Max spectroradiometer (ASD Inc., Boulder, CO, USA) under clear sky conditions within one hour of solar noon. A total of 31 and 33 trees were randomly selected within the thinned and non-thinned forests, respectively, within ~ 30 m buffer distance of the soil moisture sensors. At each tree, a series of 10 measurements with 10 replicates per measurement (total N = 100 measurements per tree) were made using a bare fiberoptic cable with a leaf clip on three different parts of the tree canopy. Reflectance was calibrated between samples using a non-calibrated diffuse white reference panel (ASD Inc., Boulder, CO). The resulting spectra ranged 350–2500 nm at ~ 1 nm spectral resolution. Four different spectral indices were calculated: (1) Water Band Index (WBI) using the 900 and 980 nm spectral bands^54^; (2) Moisture Stress Index (MSI) using the 819 nm and 1,599 nm spectral bands^55^; (3) Normalized Difference Water Index (NDWII) using the spectral bands at 857 nm and 1,241 nm^56^; and (4) Normalized Difference Infrared Index (NDII) using the 819 nm and 1,649 nm^57^. The WBI and MSI are simple band ratios that are sensitive to leaf water content changes and they both increase with increasing water stress. The NDWI and NDII are normalized band ratios that are sensitive to changes in forest canopy water content. In contrast to the WBI and MSI, these index values decrease with increasing water stress. Following Levene’s tests, all index values were compared with an ANOVA test for significant differences between the thinned (N = 31) versus non-thinned (N = 33) forest canopies.

### Forest soil moisture measurements

We installed a total of 93 Meter Terros 21 soil moisture sensors in spring 2018: 34 sensors at 25 cm in the soil profile (N = 28 in thinned forest and N = 6 in non-thinned forest), 44 sensors at 50 cm (N = 35 in thinned and N = 9 in non-thinned forest), and 15 sensors at 100 cm (N = 12 in thinned forest and N = 3 in non-thinned forest). We ensured that the sensors were distributed across the study area with comparable biophysical variability, other than the thinning treatment, in factors such as elevation, topography, soils, and fertility^34^. The sensors measured hourly soil water potential (SWP) in kilopascals (kPa) with an accuracy of 0.1 kPa and soil temperature (ST) in degrees Celsius (°C) with an accuracy of 0.1 °C (Decagon Devices, 2017) from spring 2018 through the 2021 fore-summer drought period. The fore-summer drought season is defined as the period from April 1, the end of the spring snowmelt when soil is typically near full saturation, until the onset of the North American Monsoon season, which is usually in July or August each year. The first monsoonal precipitation that ranged between 5–10 mm marked the end of the fore-summer drought period. SWP values indicate the amount of pressure required to extract water from the soil. Therefore, the more negative SWP values indicate more energy is required to extract soil water by plants.

We used two-way ANOVA tests to assess the effects of forest thinning as well as soil depth on: (a) mean soil water potential, (b) timing of critical drying onset, and (c) length of the critical drying period during the fore-summer drought season, an important time for tree water availability in the North American southwest. Time-series data from each sensor was analyzed to control for quality and unrealistic values (< −5000 kPa (METER Group Inc.)) or large data gaps due to equipment malfunction were thrown out entirely. Data from all sensors at each soil depth within thinned and non-thinned forests were then grouped together to estimate mean soil water potential. Prior to the ANOVA tests, we generated boxplots and tested for normal distributions using Levene’s test. The critical drying onset marks the date when soil water potential falls below −1000 kPa. For sensors that never reached the threshold −1000 kPa, their critical drying onset was set to the conservative value of July 13, 2021, the first day of the monsoon season. The length of the critical drying period was estimated as the number of consecutive days when soil water potential is below a critical drying threshold of −1000 kPa (CDT)^34^. When soil water potential is below the −1000 kPa threshold, significant vegetation stress is induced in ponderosa pine forests^34,44,45^. The number of consecutive days below this threshold, therefore, indicates the length of significant stress endured by the forest ecosystem.
